# Genotype–Phenotype Correlation Insights in a Rare Case Presenting with Multiple Osteodysplastic Syndromes

**DOI:** 10.3390/genes16080871

**Published:** 2025-07-24

**Authors:** Christos Yapijakis, Iphigenia Gintoni, Myrsini Chamakioti, Eleni Koniari, Eleni Papanikolaou, Eva Kassi, Dimitrios Vlachakis, George P. Chrousos

**Affiliations:** 1 Unit of Orofacial Genetics, 1st Department of Pediatrics, School of Medicine, National Kapodistrian University of Athens, “Aghia Sophia” Children’s Hospital, 115 27 Athens, Greece; iph.gintoni@gmail.com (I.G.); m.chamakioti@gmail.com (M.C.); 2 Choremion Laboratory, Research Institute of Maternal and Child Health and Precision Medicine, “Aghia Sophia” Children’s Hospital, 115 27 Athens, Greece; helenia8@yahoo.it (E.K.); papanikolaou.el77@gmail.com (E.P.); chrousge@med.uoa.gr (G.P.C.); 3 1st Department of Internal Medicine, School of Medicine, National Kapodistrian University of Athens, Laikon Hospital, 115 27 Athens, Greece; ekassi@med.uoa.gr; 4 Laboratory of Genetics, Department of Biotechnology, School of Applied Biology and Biotechnology, Agricultural University of Athens, 11855 Athens, Greece; dimvl@aua.gr

**Keywords:** osteodysplastic syndromes, skeletal anomalies, osteogenesis, bone dysplasia, bone density, Wnt pathway, genetic testing, molecular modeling

## Abstract

Background: Osteodysplastic syndromes comprise a very diverse group of clinically and genetically heterogeneous disorders characterized by defects in bone and connective tissue development, as well as in bone density. Here, we report the case of a 48-year-old female with a complex medical history characterized by bone dysplasia, hyperostosis, and partial tooth agenesis. Methods: Genetic testing was performed using WES analysis and Sanger sequencing. Molecular modeling analysis and dynamics simulation explored the impact of detected pathogenic variants. Results: The genetic analysis detected multiple pathogenic variants in genes *CREB3L1*, *SLCO2A1*, *SFRP4*, *LRP5,* and *LRP6*, each of which has been associated with rare osteodysplastic syndromes. The patient was homozygous for the same rare alleles associated with three of the identified autosomal recessive disorders osteogenesis imperfecta type XVI, primary hypertrophic osteoarthropathy, and metaphyseal dysplasia Pyle type. She also had a variant linked to autosomal dominant endosteal hyperostosis and a variant previously associated with increased risk of osteoporosis and bone fractures. Two of the detected variants are predicted to cause abnormal splicing, while molecular modeling and dynamics simulations analysis suggest that the other three variants probably confer altered local secondary structure and flexibility that may have functionally devastating consequences. Conclusions: Our case highlights the rare coexistence of multiple osteodysplastic syndromes in a single patient that may complicate differential diagnosis. Furthermore, this case emphasizes the necessity for early genetic investigation of such complex cases with overlying phenotypic traits, followed by genetic counseling, facilitating orchestration of clinical interventions and allowing prevention and/or prompt management of manifestations.

## 1. Introduction

Osteodysplastic syndromes are a group of more than 770 clinically and genetically heterogeneous disorders characterized by defects in bone and connective tissue development, which may include skeletal dysplasias and systemic deformities, representing about 5% of newborns with congenital malformations [1]. They comprise a very diverse group of disorders including short stature conditions due to abnormalities of bone development such as in achondroplasia, decreased bone density as in osteogenesis imperfecta, or increased bone density as in osteopetrosis [1]. They affect up to 20 in 100,000 neonates, most of whom exhibit a genetic background associated with pathological variants in more than 550 genes [2]. The complete spectrum of inherited skeletal disorders has been classified into 42 groups, based on molecular, biochemical, and/or radiographic criteria [3].

Skeletal dysplasias may be inherited in an autosomal dominant, autosomal recessive, or X-linked manner. The pathophysiology of skeletal dysplasias lies in the disruption of cellular pathways, which are important in bone growth, patterning, and mineralization [4,5,6]. Such pathways include the Indian Hedgehog (IHH) pathway, the fibroblast growth factor (FGF) pathway, the transforming growth factor beta (TGF-β) pathway, as well as the canonical and noncanonical Wingless-related integration site (Wnt) pathway [7]. The genes implicated in skeletal dysplasias account for bone growth, cartilage formation, and extracellular matrix development. Pathological variants of the same gene may cause different skeletal dysplastic conditions [3]. In contrast, one form of skeletal dysplasia may be triggered by many different genes [8].

Typically, skeletal dysplasias are evident in childhood, but they can also manifest as early as the prenatal period, or as late as adolescence. However, atypical cases have been recorded as well, with a presentation throughout adulthood. An example of an atypical presentation is the one of a 25-year-old woman who experienced severe lower back pain and walking difficulties postpartum two weeks after delivery. Radiological examinations revealed severe osteoporosis. Despite a positive history of multiple childhood fractures, this adult-onset presentation was atypical for osteogenesis imperfecta, highlighting the disorder’s potential variability [9].

Due to their often atypical presentation and complex genetic background, diagnosing skeletal dysplasias is a complex process through which clinical, radiological, and genetic results are evaluated. This process has evolved over the years. While the approach was initially based on clinical and imaging information, today, high-technology molecular genetic techniques are used to identify the genetic basis of the disorders. Widely used techniques include whole-exome or whole-genome next-generation sequencing (NGS), whole-genome single-nucleotide polymorphism mapping, comparative genomic hybridization arrays, and targeted enrichment of a locus followed by next-generation sequencing (NGS) [2,7,10].

Here we present a patient with a complex medical history characterized by bone dysplasia, hyperostosis, and dental agenesis. Genetic testing revealed multiple pathogenic variants associated with rare osteodysplastic syndromes and molecular modeling analysis revealed the disruption of mutant protein structures and functions caused by the observed pathogenic variants. In view of the present findings, we briefly but critically review the published knowledge of the relevant osteogenesis and bone-remodeling mechanisms.

## 2. Materials and Methods

### 2.1. Data Collection

The study involved an individual of Greek descent with skeletal dysplasia, hyperostosis, and partial anodontia. The investigation was ethically approved by the Bioethics Committee of the University Research Institute for the Study of Genetic and Malignant Disorders in Childhood at the School of Medicine of the National Kapodistrian University of Athens (RPURI9002). After thorough genetic counseling and signed informed consent, the patient provided a whole-blood sample for molecular genetic analysis to take place, in order to determine the exact underlying genotype(s).

### 2.2. DNA Sequence Analysis

Genomic DNA was extracted from the patient’s white blood cells using Nucleospin^®^ Blood Quickpure kit (Macherey Nagel GmbH, Düren, Germany). Whole-exome sequencing (WES) was performed using DNBSEQ-G400 (MGI Tech, Shenzhen, Guangdong, China). The DNA was analyzed using a hybridization-based target enrichment method (KAPA HyperExome Probes, 43 Mb, Roche, Indianapolis, IN, USA). The targeted regions include exons and adjacent intronic sequences of the analyzed genes. This was followed by a bioinformatic analysis of the obtained DNA sequences and comparison with a reference sequence (GRCh37). The potential impact of missense variants on protein structure and function was assessed using the MetaSVM 0.5.2 consensus prediction algorithm (PMID: 25552646), which integrates the results of ten prediction algorithms—SIFT, PolyPhen-2 HDIV, PolyPhen-2 HVAR, GERP++, MutationTaster, Mutation Assessor, FATHMM, LRT, SiPhy, and PhyloP (doi:10.1093/bioinformatics/btv204, assessed on 14 March 2025)—to calculate a score ranging from −2 to 3. The mean sequencing coverage depth was 100X, with 98% of target regions sequenced at a depth of ≥20X. Targeted DNA sequencing of the genomic regions containing the pathological variants was performed by using the automated capillary sequencer ABI 3730 XL Analyzer (Applied Biosystems, Waltham, MA, USA).

### 2.3. Molecular Modeling and Molecular Dynamics Simulations

In order to explore the structural consequences of pathological variants we employed an integrative in silico approach, combining homology modeling and molecular dynamics simulations for those proteins without an available X-ray structure. The goal was to examine how these single-amino acid substitutions might alter the local structure, protein stability, or dynamics within the Wnt signaling pathway.

Homology models for both wild-type and mutant proteins were generated using MODELLER (https://salilab.org/modeller/ accessed on 5 May 2025). Full-length sequences of the human proteins were obtained from the UniProt database, and specific domains containing or surrounding the mutation sites, were prioritized for modeling based on functional annotations and the availability of homologous structures. Suitable template structures were identified using BLASTp and HHpred searches against the Protein Data Bank (PDB), favoring those with sequence identities above 30% and resolutions better than 3.0 Å. Sequence-to-structure alignments were refined to preserve secondary structural features, and in each case, the mutant residue was introduced at the sequence level prior to model building to allow MODELLER to optimize side-chain packing during structure generation. For each construct, 100 models were generated, and the one with the lowest DOPE score was selected for further refinement and analysis. Structural integrity and stereochemical quality of the final models were evaluated using PROCHECK PROCHECK (https://www.ebi.ac.uk/thornton-srv/software/PROCHECK/ accessed on 12 May 2025), MolProbity (https://molprobity.biochem.duke.edu accessed on 12 May 2025), and Verify3D (https://www.doe-mbi.ucla.edu/verify3d/ accessed on 12 May 2025).

To assess the effect of each mutation on protein dynamics and conformational stability, we conducted atomistic molecular dynamics simulations using GROMACS (https://www.gromacs.org/ accessed on 13 May 2025), applying the CHARMM27 force field. Each modeled structure was placed in a periodic dodecahedral simulation box filled with TIP3P water molecules, ensuring at least a 1.0 nm buffer between the protein surface and the box boundary. The systems were neutralized with Na^+^ or Cl^−^ ions and adjusted to a physiological ionic strength of 0.15 M NaCl.

Prior to simulation, each system underwent energy minimization, using the steepest descent algorithm to remove steric clashes and optimize initial geometry. Equilibration was performed in two phases: a constant volume (NVT) simulation at 310 K for 100 ps, followed by a constant pressure (NPT) phase at 1 bar for another 100 ps. During equilibration, position restraints were applied to the heavy atoms of the protein to maintain structural integrity while allowing solvent relaxation. Unrestrained production simulations were subsequently run for 100 ns with a 2 fs integration time step. The LINCS algorithm was used to constrain all bonds involving hydrogen atoms, and long-range electrostatics were computed using the Particle Mesh Ewald (PME) method with a cutoff of 1.2 nm for short-range interactions.

Trajectory data were analyzed using built-in GROMACS tools (https://www.gromacs.org/ accessed on 13 May 2025), Backbone root mean square deviation (RMSD) and per-residue root mean square fluctuation (RMSF) were calculated to assess global and local structural flexibility, respectively. The radius of gyration (Rg) was computed to track compactness over time, while hydrogen bond analysis and solvent-accessible surface area (SASA) were used to evaluate structural rearrangements and solvent exposure. Additionally, secondary-structure evolution was monitored using the DSSP algorithm. Visualization and qualitative inspection of protein trajectories were performed with VMD-Visual Molecular Dynamics (https://www.ks.uiuc.edu/Research/vmd/ accessed on 13 May 2025) and PyMOL (https://pymol.org/ accessed on 13 May 2025), allowing us to directly compare conformational behavior between wild-type and mutant forms.

This modeling and molecular dynamics framework provided a detailed view of the potential mechanistic impact of each mutation at the atomic level, enabling interpretation of their possible functional roles within the context of Wnt signaling.

## 3. Results

### 3.1. Case Report

**Figure 1:**
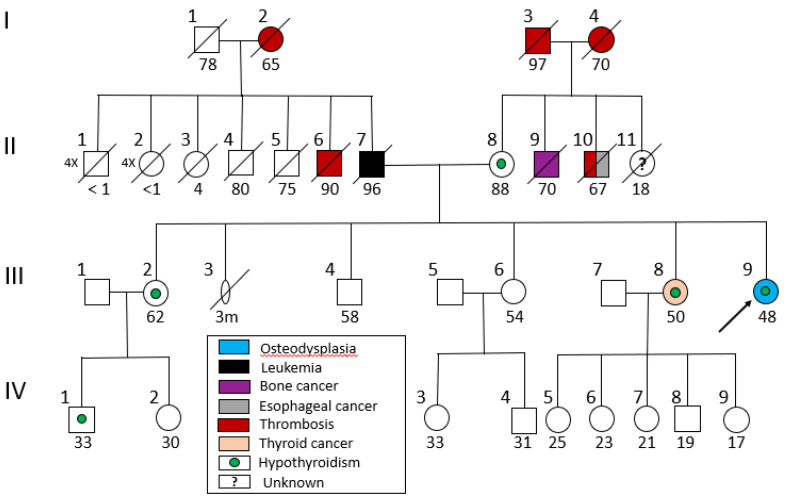
Pedigree of the patient’s family. The patient (III-9) is shown with an arrow. Light blue color indicates osteodysplastic syndrome and other colors correspond to various disorders.

A 58-year-old female with no family history of skeletal dysplasia was referred by an endocrinologist. She was the fifth-born child of parents that originated from different villages of the same prefecture in Central Greece (Figure 1). The family history, spanning four generations (I-IV), revealed cases of hypothyroidism and various cancers, including leukemia in the father of the patient’s maternal grandmother, thyroid cancer (papillary carcinoma) in a maternal aunt, and multiple instances of cancer in other distant family members. There was no indication of consanguinity between the patient’s parents.

**Figure 2:**
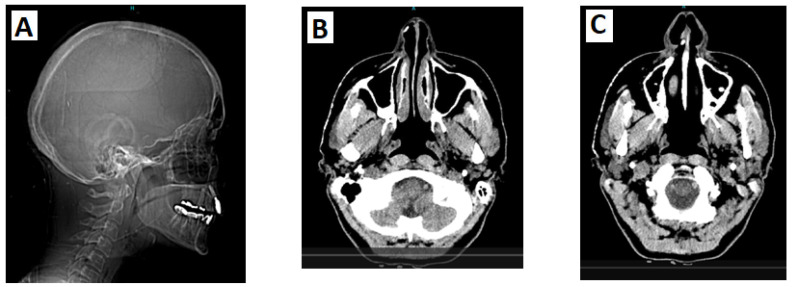
(**A**) CT scan of the head and neck (sagittal plane) reveals a bony projection near the anterior border of the right maxillary sinus, whereas the inferior turbinate shows slight calcification/thickening; (**B**) CT brain angiography shows a focal calcification in the lateral cartilaginous wall of the right inferior nasal cavity; (**C**) CT brain angiography reveals an osteophytic spur at the anterior border of the right maxillary sinus that projects into the right nasal cavity.

**Figure 3:**
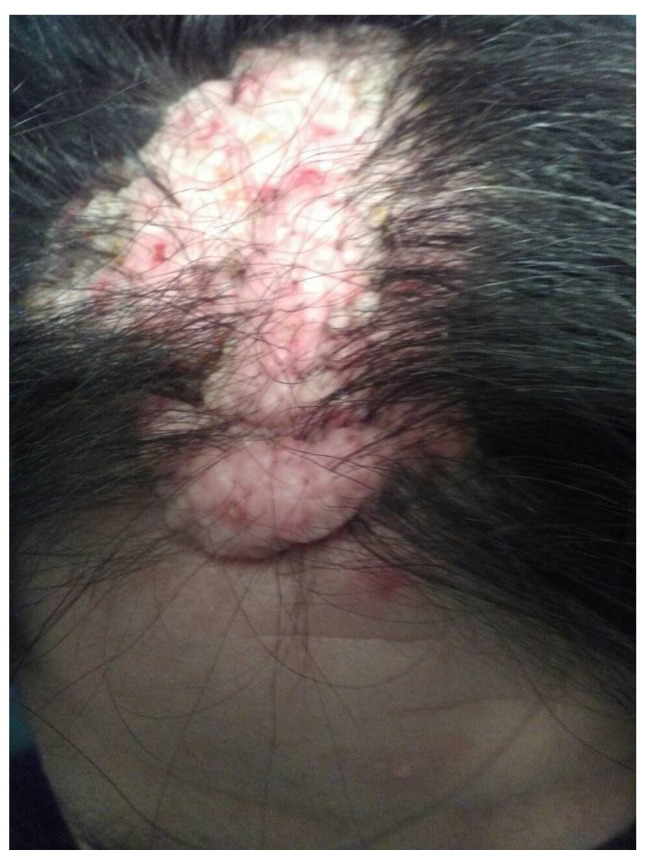
Benign intradermal melanocytic nevus on patient’s forehead skin. A biopsy revealed osteoma and bony metaplasia of a benign chorionic nevus.

The patient first reported missing teeth (right side oligodontia) at the age of 8 years. At 18, she was diagnosed with hypothyroidism. A year later she noticed the development of hyperostosis and bony malformations on the right side of her face, including her nose, forehead, skull, and below the right eye (Figure 2). Over the years, she underwent multiple maxillofacial surgeries to address impacted teeth and had two skin biopsies, which revealed osteoma and bony metaplasia of a benign chorionic nevus (Figure 3). The patient also underwent radiological investigations, including a computerized tomography scan of the skull, which showed a pedunculated exostosis/osteoma in the right frontal sinus, smooth hypertrophy of the right nasal bone with microcalcifications, focal calcifications in the right inferior nasal cavity, and microcalcifications in the right temporal and frontal subcutaneous tissues (Figure 2). Brain imaging was normal.

**Figure 4:**
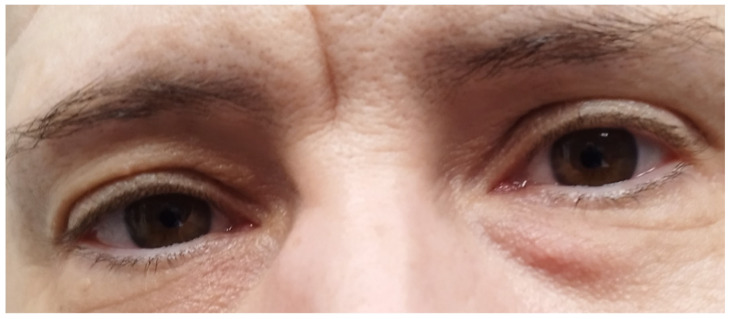
Light blue sclerae (a sign of mild osteogenesis imperfecta).

**Figure 5:**
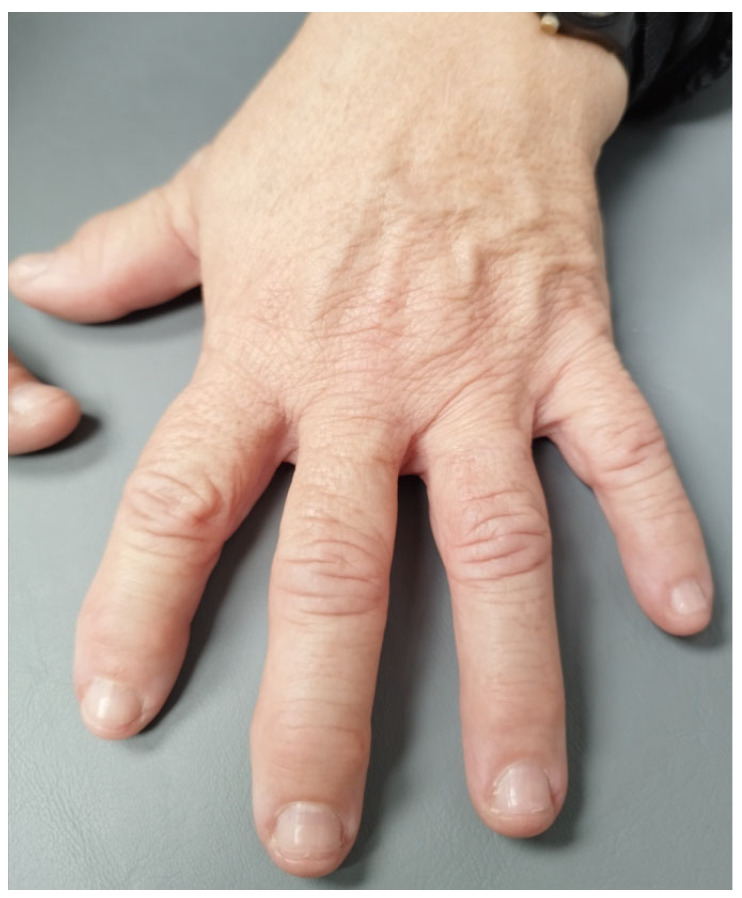
Hand with mild hypertrophic osteoarthropathy.

Physical examination of the patient revealed widening of the frontal bone and lengthening of the lower jaw, light blue sclerae (Figure 4), as well as facial acne. It also revealed a small area of bony malformation in the right frontal side of her face in contact with her hair and multiple skin microcalcifications of minimal size at the maxillary right side of the face. The patient had characteristics of an osteodysplastic syndrome, partial oligodontia and mildly hypertrophic osteoarthropathic hands (Figure 5). During genetic counseling she was advised that molecular genetic analysis was needed to determine the exact underlying gene variation(s) contributing to her phenotype(s).

In the last three years, under our care, the patient received one yearly intravenous infusion of Aclasta (zoledronic acid) for her osteoporosis. Zolendronic acid is a bisphos-phonate which binds hydroxyapatite in bone and accumulates at sites of bone remodeling. It then integrates in the osteoclasts and inhibits the breakdown of bone matrix. Although zolendronic acid did not target the genetic defects of our patient, it increased ossification and improved her clinical image by enhancing bone density and structural integrity. Since then, she has had no further exostoses/osteomas or other novel osteodysplastic manifestations. We plan routine reassessments of risk of fracture by monitoring bone mineral density every two years with dual-energy X-ray absorptiometry maintenance of adequate levels of vitamin D and calcium intake.

### 3.2. Genetic Analysis

Whole-exome sequencing of the patient’s genomic DNA interestingly revealed five pathogenic variants (four of them novel), which are associated with osteodysplastic syndromes. The detected pathological variants were verified by targeted DNA sequencing of the gene regions containing them. The patient was homozygous for the following (Table 1):

(a) A single-nucleotide deletion c.1523+3G>- in intron 11 of gene *CREB3L1* (NM_052854) on chromosome 11p11.2, predicted to cause abnormal splicing. Mutations in gene *CREB3L1* are associated with autosomal recessive osteogenesis imperfecta type XVI (OMIM 616229);
(b) A single-nucleotide substitution c.397+10T>C in intron 3 of gene *SLCO2A1* (NM_005630) on chromosome 3q22.1-q22.2, predicted to cause abnormal splicing. Mutations in gene *SLCO2A1* are associated with autosomal recessive primary hypertrophic osteoarthropathy (OMIM 614441);
(c) A single-nucleotide substitution c.958C>A in exon 6 of gene *SFRP4* (NM_003014) on chromosome 7p14.1, predicted to cause a missense substitution of proline into threonine in codon 320. Mutations in gene *SFRP4* are associated with autosomal recessive metaphyseal dysplasia Pyle type (OMIM 265900).

The patient was heterozygous for the following (Table 1):

(a) A single-nucleotide substitution c.1913G>A in exon 17 of gene *LRP5* (NM_001291902) on chromosome 11q13.4, predicted to cause a missense substitution of arginine into histidine in codon 638. Mutations in gene *LRP5* are associated with autosomal dominant endosteal hyperostosis, also known as osteosclerosis (OMIM 144750), a disorder that is compatible with the patient’s phenotype. Other allelic mutations in gene *LRP5* cause disorders with clinical characteristics not found in the patient, such as bone mineral density variability type 1 (OMIM 601884), osteopetrosis type 1 (OMIM 607634), exudative vitreoretinopathy type 4 (OMIM 601813), osteoporosis-pseudoglioma syndrome (OMIM 259770), and polycystic liver disease type 4 with or without kidney cysts (OMIM 617875).
(b) A single-nucleotide substitution c.3184A>G in exon 14 of gene *LRP6* (NM_002336) on chromosome 12p13.2, which was predicted to cause a missense substitution of isoleucine into valine in codon 1062. This variation has been associated with osteoporosis and an increased risk of fractures [11]. Other allelic mutations in the *LRP6* gene have been reported to cause autosomal dominant tooth agenesis type 7 (OMIM 616724) and autosomal dominant coronary artery disease type 2 (OMIM 610947).

### 3.3. Molecular Dynamics Simulations of Detected Pathological Variants

Two of the detected variants (c.1523+3G>- in *CREB3L1* and c.397+10T>C in *SLCO2A1*) were predicted to cause abnormal splicing. The other three predictively pathological missense variants were studied further by an integrative in silico approach commonly used for proteins without an available X-ray structure. We explored the structural consequences that the detected variants (all of them single amino acid substitutions) confer to the local structure, protein stability, or kinetics by combining molecular homology modeling and molecular dynamics simulations.

**Figure 6:**
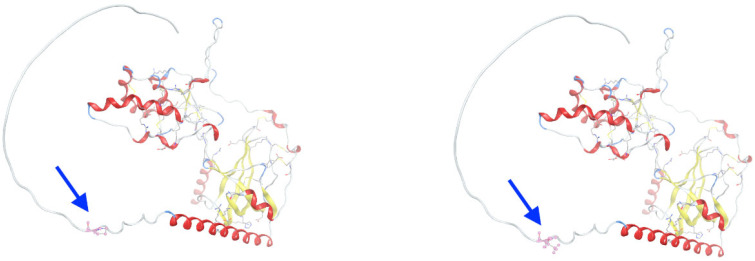
Structural comparison of wild-type and P320T-mutant SFRP4 protein. Ribbon diagrams of the C-terminal domain of SFRP4 are shown for the wild-type (**left**) and P320T mutant (**right**). The proline, a nonpolar amino acid, at position 320 (P320) in the wild-type protein is replaced by threonine (T320), a polar-uncharged amino acid, in the mutant model, as indicated by blue arrows. This residue lies near the presumed protein–protein interaction surface and may influence local electrostatic interactions and solvent accessibility. Hydrogen bonding patterns in the vicinity of the mutated residue are indicated with dashed lines. Structural overlays reveal subtle alterations in side-chain orientation and local loop flexibility.

The c.958C>A variant in gene *SFRP4* causes replacement of a proline (P320) in the wild-type protein SFRP4 by threonine (T320) at position 320, which lies near the presumed protein–protein interaction surface and may influence local electrostatic interactions and solvent accessibility (Figure 6). Structural overlays reveal subtle alterations in side-chain orientation and local loop flexibility.

**Figure 7:**
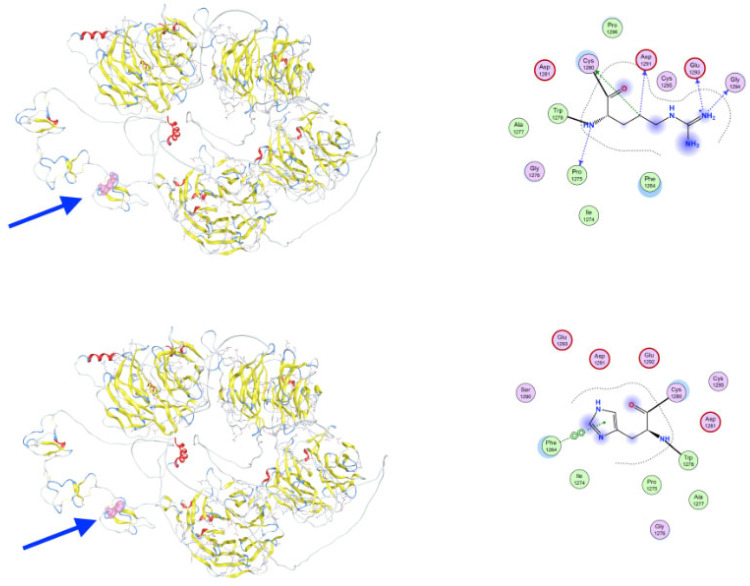
Structural impact of the R638H mutation in the extracellular domain of LRP5. Cartoon representation of the second β-propeller-EGF-like domain of LRP5, comparing wild-type R638 (**upper**) and H638-mutant (**lower**) forms. The mutated residue is shown in stick representation and its position is indicated by the blue arrow. The histidine substitution introduces a residue with altered protonation dynamics, which may disrupt hydrogen bonding and ligand-interaction surfaces. Local conformational differences are highlighted, and solvent-accessible surface area (SASA) mapping indicates increased exposure in the mutant form.

The c.1913G>A variant in gene *LRP5* causes substitution of an arginine (R638) in the wild-type protein LRP5 into histidine (H638) at position 638 in the extracellular domain of LRP5. The histidine substitution introduces a residue with altered protonation dynamics, which may disrupt hydrogen-bonding and ligand-interaction surfaces (Figure 7). Local conformational differences are highlighted, and solvent-accessible surface area (SASA) mapping indicates increased exposure in the mutant form.

**Figure 8:**
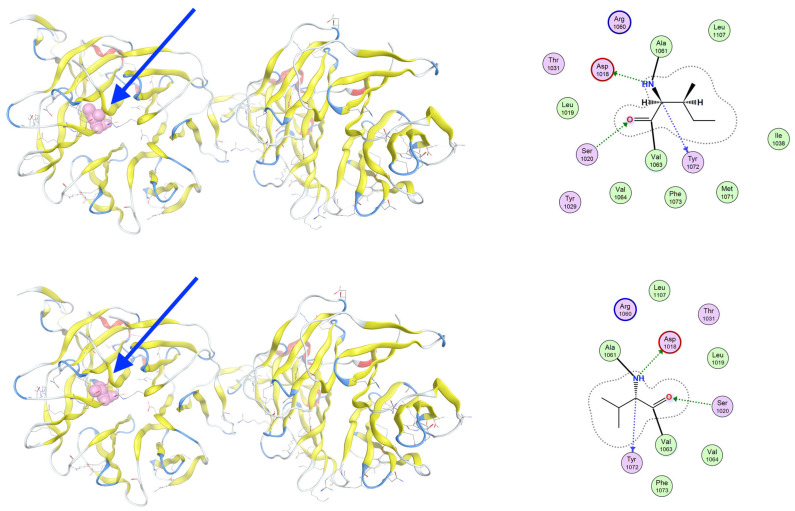
Structural comparison of wild-type and I1062V-mutant LRP6 intracellular domain. Overlay of wild-type (**upper**) and mutant (**lower**) LRP6 intracellular domains, with a focus on the region surrounding residue 1062, as indicated by the blue arrow. The mutation I1062V, although conservative, introduces a change in side-chain volume that may affect the packing of adjacent residues. The position of the mutation is marked in red. Local secondary structure remains largely preserved, but molecular dynamics simulations suggest altered flexibility and transient exposure of nearby signaling motifs.

The c.3184A>G variant in the *LRP6* gene causes the substitution of isoleucine by valine at position 1062 (I1062) of the wild-type LRP6 protein (V1062). The mutation I1062V, although conservative, introduces a change in side-chain volume that may affect the packing of adjacent residues. Although the local secondary structure remains largely preserved, molecular dynamics simulations suggest altered flexibility and transient exposure of nearby signaling motifs (Figure 8).

## 4. Discussion

Osteodysplastic syndromes comprise a very diverse group of clinically and genetically heterogeneous disorders characterized by defects in bone and connective tissue development as in achondroplasia, decreased bone density as in osteogenesis imperfecta, or increased bone density as in osteosclerosis [1,2,3,4,12,13,14]. The patient presented here has had a complex medical history characterized by bone dysplasia, hyperostosis, and partial dental agenesis. Genetic testing revealed multiple pathogenic variants associated with rare osteodysplastic syndromes. Apparently, the patient exhibits autosomal recessive osteogenesis imperfecta type XVI (OMIM 616229), autosomal recessive primary hypertrophic osteoarthropathy (OMIM 614441), autosomal recessive metaphyseal dysplasia Pyle type (OMIM 265900), autosomal dominant endosteal hyperostosis (OMIM 144750), as well as increased risk of osteoporosis and fractures.

Notably, the fact that the patient is homozygous for the same rare alleles associated with three of the identified autosomal recessive disorders points to the potential for consanguinity between the parents. Although no such relationship was identified in the family history, a common ancestor of the patient’s parents cannot be ruled out in the light that they originated from relatively close villages of the same Prefecture in Central Greece.

### 4.1. CREB3L1 in Osteogenesis Imperfecta Type XVI

The patient is homozygous for the novel single-nucleotide deletion c.1523+3G >- in intron 11 of gene *CREB3L1* (11p11.2), which is predicted to cause abnormal alternative splicing and most probably lead to reduced levels of functional CREB3L1, thus contributing to the patient’s relatively mild compatible phenotype of autosomal recessive osteogenesis imperfecta type XVI. She has light blue sclerae, apparently unmarked demineralization or decrease in skull ossification, and reportedly no multiple fractures of ribs and long bones. Autosomal recessive osteogenesis imperfecta type XVI is characterized by reduced ossification of the skull, osteopenia, as well as by an increased risk of fractures. The gene *CREBL1* encodes a transcription factor (cAMP responsive element-binding protein-like 1) which activates the mechanism against the badly folded proteins in the endoplasmic reticulum. As a result of the malfunction of the CREB3L1 protein, there is reduced ossification of the skull. In this patient, the homozygous *CREB3L1* mutation affects splicing, and likely results in reduced functional-protein production, but does not lead to the complete absence of CREB3L1 [15].

**Figure 9:**
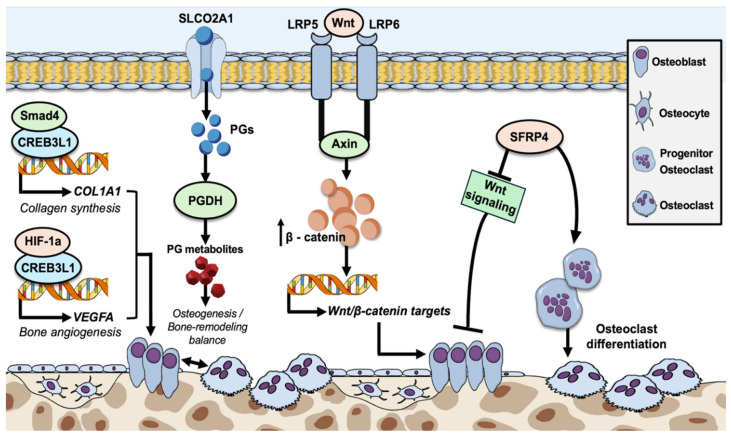
Molecular mechanisms of osteogenesis and bone remodeling, through osteoblast proliferation and/or inhibition of osteoclast differentiation, or by induction of osteoblast formation, respectively. CREB3L1, abundantly expressed in osteoblasts, interacts with Smad4, which facilitates its translocation to the nucleus, where it promotes the expression of the *COL1A1* gene, resulting in the production of collagen type I α1 chain, essential for collagen formation, the predominant component of bone extracellular matrix. CREB3L1 also interacts with HIF-1a, subsequently leading to the synergistic upregulation of *VEGFA* gene expression, which is in turn crucial for oxygen and nutrient provision in bone tissue. SLCO2A1, a prostaglandin anion transmembrane transporter, facilitates the intracellular transfer of prostaglandins (mainly of PGE2) for subsequent metabolism by PGDH, thus regulating their levels and preventing accumulation. Prostaglandins are key factors for osteogenesis, mainly by stimulating osteoblast activation, while simultaneously inhibiting osteoclast development. Their balanced levels, highly reliant on normal SLCO2A1 function, are crucial for the maintenance of a normal osteogenesis/bone-remodeling ratio. LRP5 and LRP6 both act as membrane co-receptors of the Wnt/β-catenin pathway that contribute to the enhancement of signal transduction through the binding of Wnt ligands, which induces the phosphorylation of their carboxy-terminal ends, followed by the recruitment and binding of Axin. This subsequently leads to the disruption of β-catenin’s destruction complex and therefore its nucleic and cytoplasmic accumulation, thus facilitating the enhanced transcription of Wnt/β-catenin downstream target-genes. Finally, the SFRP4 glycoprotein negatively regulates osteogenesis through the inhibition of both osteoblast proliferation and differentiation, as well as the simultaneous induction of osteoclast differentiation through the inhibition of Wnt signaling.

CREB3L1 (also known as is OASIS) is highly expressed in osteoblasts (Figure 9) and plays a pivotal role in osteogenesis mainly through the expressional regulation of key implicated genes [16,17,18]. CREB3L1 is known to activate the transcription of gene *COL1A1*, coding for the collagen type 1 α1 chain, which is pivotal for collagen production, the major component of bone extracellular matrix [18,19]. This takes place in light of chronic TGF-β stimulation, which induces CREB3L1 to bind to Smad4 and therefore enables its translocation to the nucleus, which in turn directly activates the transcription of *COL1A1* [17]. The role of CREB3L1 also extends to bone angiogenesis, through its significant rise in response to hypoxia, followed by its interaction with the hypoxia-inducible factor-1α (HIF-1α), finally leading to the synergistic upregulation of VEGFA, thus resulting in oxygen and nutrient supply in bone tissue [17,18].

Both normal availability and functionality of CREB3L1, are proven to strongly determine bone density and structural integrity. In cases of variants that affect the *CREB3L1* gene’s expression or its protein product’s CREB3L1 binding capacity on transcriptional targets, they might lead to abnormal collagen synthesis and result in moderate or severe osteogenesis imperfecta [16,20]. Additionally, it has been demonstrated that mutations in the *CREB3L1* gene are associated with hypodontia and oligodontia [21].

### 4.2. SLCO2A1 in Primary Hypertrophic Osteoarthropathy

Furthermore, the patient is homozygous for the novel single-nucleotide substitution c.397+10T>C in intron 3 of the *SLCO2A1* gene (3q22.1-q22.2), also predicted to cause abnormal splicing and possibly accounting for reduced production of the encoded protein. The signs of autosomal recessive primary hypertrophic osteoarthropathy in the patient include periosteal inflammation and osteoarthropathy, variable features of pachydermia, pedunculated exostosis/osteoma in the right frontal sinus, smooth hypertrophy of the right nasal bone with microcalcifications, focal calcifications in the right inferior nasal cavity, bony metaplasia of frontal benign chorionic nevus, and microcalcifications in the right temporal and frontal subcutaneous tissues.

The *SLCO2A1* gene encodes a solute carrier organic anion transporter 2A1 (SLCO2A1) that facilitates the transport and degradation of prostaglandins (Figure 9), primarily regulating the levels of prostaglandin E2 (PGE2). Prostaglandins are highly implicated in osteogenesis, as well as bone metabolism and homeostasis, mainly by stimulating the osteoblast activation, while inhibiting osteoclast development, thus contributing to the preservation of bone density and skeletal integrity [22,23]. The aberrant function of SLCO2A1 due to missense mutations in its coding gene might lead to impaired transport and degradation of PGE2, thus resulting in its pathological systemic accumulation [23]. Elevated PGE2 levels have been shown to induce abnormalities of bone remodeling and bone inflammation, which have been associated with a rare autosomal recessive primary hypertrophic osteoarthropathy [22,24,25,26,27].

### 4.3. SFRP4 in Metaphyseal Dysplasia/Pyle’s Disease

The patient is homozygous for the novel single-nucleotide substitution c.958C>A in exon 6 of the *SFRP4* gene (7p14.1), predicted to cause a missense substitution of proline into threonine in codon 320. Although our patient’s clinical picture is relatively compatible with the phenotype of autosomal recessive metaphyseal dysplasia Pyle type, it is indeed milder than most affected individuals who usually present with nonsense variants. Typical Pyle’s disease is marked by dysplasia of the outer part of the bones that are thinner than normal (flattened form), delayed eruption of permanent teeth, broad metaphyses of long bones, and susceptibility to fractures [28,29,30,31,32]. The signs of the rare disorder in our patient include a mildly affected skull, partial tooth agenesis, and a partially thin cortical bone.

The *SFRP4* gene codes for secreted frizzled-related protein 4 (SFRP4), an extracellular inhibitor of the intracellular Wnt-signaling pathway, which acts by directly binding Wnt ligands and preventing their interaction with Frizzled/LRP5/6 receptor complexes (Figure 9). Through its antagonistic role against the Wnt pathway, SFRP4 negatively regulates osteogenesis through the inhibition of both osteoblast proliferation and differentiation, as well as the regulation of osteoclast differentiation, which are essential parts of osteogenesis and bone homeostasis [30,33]. Induced *SFRP4* knockout in osteoclasts has been shown to induce the activation of the Wnt/β-catenin pathway, as well as abnormal activation of Wnt/Ror2/Jnk signaling, thus leading to increased osteoblast differentiation and inhibition of osteoclast differentiation, respectively [33].

The novel P320T mutation detected in homozygosity in our patient represents a non-conservative substitution of a nonpolar proline residue with a polar uncharged threonine near the C-terminal region of the protein. This domain is thought to participate in protein–protein and protein–matrix interactions, and such a change might affect the folding, stability, and/or secretion of SFRP4. Functionally, diminished SFRP4 activity could result in unchecked Wnt signaling activation, potentially altering cellular proliferation and differentiation. Elevated Wnt activity has been implicated in the pathogenesis of several malignancies, while reduced *SFRP4* expression has been associated with insulin resistance and abnormal bone remodeling. Although P320T has not been previously reported in major variant databases, its biochemical characteristics and location suggest it may be functionally disruptive.

### 4.4. LRP5 in Endosteal Hyperostosis/Osteosclerosis

The patient is heterozygous for a novel single-nucleotide substitution c.1913G>A in exon 17 of gene *LRP5* (11q13.4), predicted to cause a missense substitution of arginine into histidine in codon 638. Mutations in the *LRP5* gene are associated with autosomal dominant endosteal hyperostosis (also known as osteosclerosis), which is characterized by generalized cortical thickening of long bones, without alteration in external shape, which confers remarkable bone resistance to fracture [34]. It is also characterized by craniofacial bone growth, such as widening of the frontal bone and lengthening of the lower jaw during adolescence (observed in our patient). The identified mutation likely contributes to generalized increased bone density that probably has rendered milder the signs of osteogenesis imperfecta or the risk for osteoporosis and fractures that variant I1062V in gene *LRP6* confers.

LRP5 (low-density lipoprotein receptor-related Protein 5), similarly to LRP6, serves as a co-receptor that enhances the signaling within the Wnt/β-catenin pathway (Figure 9), which is crucial for normal postnatal bone formation and plays a key role in osteoblast activity [35,36]. In addition, increased *LRP5* expression in cartilage is linked to the pathogenesis of osteoarthritis as well as concurrent osteoporosis [37,38].

The novel R638H mutation detected in our patient affects a conserved arginine residue within the second β-propeller-EGF-like module of the extracellular domain of LRP5. This domain is crucial for ligand binding and proper folding of the receptor. Arginine-to-histidine substitutions, while partially conserved in charge, can have context-dependent effects due to the unique pKa and hydrogen bonding potential of histidine. LRP5 is essential for skeletal development, and both loss-of-function and gain-of-function mutations in its gene are associated with bone mineral density disorders. Importantly, several mutations near position 638 have been implicated in autosomal recessive osteoporosis-pseudo-glioma syndrome and high bone-mass phenotypes, suggesting that R638H may similarly affect the receptor’s affinity for Wnt ligands or its ability to form functional receptor complexes. Additionally, given the role of LRP5 in glucose metabolism, via osteocalcin signaling, this variant may influence metabolic traits beyond skeletal phenotypes.

### 4.5. LRP6 Associated with Osteoporosis and an Increased Risk of Fractures

The patient is heterozygous for the single-nucleotide substitution c.3184A>G in exon 14 of the *LRP6* gene (12p13.2), predicted to cause a missense substitution of isoleucine by valine in codon 1062. This variant has been associated with osteoporosis and an increased risk of fractures [11,39]. In our patient this variant has probably antagonized and rendered milder the signs of generalized increased bone density due to the R638H variant in *LRP5* which is associated with autosomal dominant endosteal hyperostosis. Our patient also suffered from missing teeth and angulations during the eruption of teeth, and several missense variants in gene *LRP6* have been associated with tooth agenesis, but not the I1062V variant. As mentioned above, partial tooth agenesis in our patient probably is due to the P320T variant in gene *SFRP4*.

The *LRP6* gene codes for low-density lipoprotein receptor-related protein 6 (LRP6), which is a membrane co-receptor together with proteins of the frizzled family to activate the intracellular the Wnt/β-catenin pathway that enhances signal transduction through the internalization of Wnt receptors (Figure 9). Following the appropriate ligand binding, it ultimately results in osteoblast activity as well as in proliferation and differentiation of dental epithelial and mesenchymal cells [40,41,42,43].

The I1062 is highly conserved during evolution in Xenopus frogs, chicken, mice, and humans; therefore, it has an important role in protein function. Isoleucine is always present at the same position in the third blade of the first, second, and fourth β-propeller structure of both six-bladed LRP6 and LRP5. The same structure pattern that includes isoleucine at that same position is found in other proteins with a β-propeller domain including epidermal growth factor precursor, very-low-density lipoprotein receptor, and other LDL receptor-related proteins [11].

The I1062V substitution in LRP6 involves a conservative isoleucine-to-valine change within the intracellular domain of the co-receptor. Although structurally similar, such substitutions can still impact local hydrophobic interactions or modulate post-translational modifications, particularly if occurring near key signaling motifs. LRP6 plays a critical role in the canonical Wnt/β-catenin pathway and has been linked to impaired bone accrual as well as early-onset cardiovascular disease and metabolic syndrome. Variants in the intracellular region of LRP6 can affect phosphorylation-dependent signal propagation or alter its interaction with the cytoplasmic phosphoprotein Disheveled that acts directly downstream of frizzled receptors. While the I1062V mutation has not been extensively studied, analogous substitutions in the LRP6 intracellular tail have shown altered downstream signaling, reinforcing the potential relevance of this variant in pathological phenotypes.

### 4.6. Wnt Signaling Pathway Variants

Interestingly, in this study, we identified and characterized three missense variants affecting key components of the Wnt signaling pathway: P320T in *SFRP4*, and I1062V and R638H in *LRP5* and in *LRP6*. These alterations might have significant implications for the regulation of Wnt-mediated processes, including bone metabolism, glucose homeostasis, and tissue remodeling (Table 2). All in all, these three mutations converge on components of the Wnt signaling pathway and underscore the delicate balance between activation and inhibition that governs developmental and homeostatic processes. While the precise phenotypic outcomes of each variant remain to be fully elucidated, their locations in structurally and functionally critical regions strongly suggest potential pathogenicity or physiological relevance. Future studies employing structural modeling, Wnt reporter assays, and animal models will be essential to validate these hypotheses and to determine the broader implications of these variants in health and disease.

### 4.7. Limitations of the Study

Despite the fact that the present complex case was thoroughly and multidimensionally investigated, several factors might limit the generalizability of the findings and the patient’s phenotypic evaluation. The low prevalence of such cases of complex osteodysplastic phenotypes does not usually allow for the conduction of large-scale, systematic studies within the same hospital or research center, thus limiting the findings and their interpretation to singular cases, without similar data-sets to compare and produce generalizable conclusions and deeply understand the interplay between genetic and clinical heterogeneity. Furthermore, some of the gene variants reported here are novel and were assessed through bioinformatics analysis only. Acknowledging the limitations they pose for reproducibility, experimental validation is recommended to strengthen the interpretation.

The presence of five distinct mutations across different genes, associated with osteogenic disorders, within a single patient with such a complex phenotype, renders the interpretation of findings an exceedingly challenging matter. Such complexity arises due to the fact that epistatic interactions remain unclear, and despite the fact that the impact of each mutation on the respective protein has been elucidated, the individual contribution of each variant to the patient’s phenotype can only be hypothesized based on the available clinical and molecular data. Additionally, the fact that the patient was clinically approached as a complex case, and was hence referred for genetic counseling at that age, has led to the absence of critical medical documentation from the initial stages of each clinical manifestation, thus making it difficult to “decode” and interpret her current mixed clinical picture, or map the phenotypic course.

### 4.8. Potential Future Prospects of the Study

The present case suggests that behind each clinical phenotype of osteodysplasia, there may be more than one genetic defect, possibly even a unique combination of genetic mutations. Therefore, in-depth clinical investigation and early genetic testing may be necessary for accurate diagnosis and proper treatment of such complex cases. Detailed imaging of bone structure specialized for osteodysplasias may include high-resolution computed tomography, whereas quantitative computed tomography may accurately measure cortical and trabecular bone density.

Cases of coexistence of multiple osteodysplastic syndromes in a single patient might be infrequent, yet they present in the general population, and a number of them might be overlooked due to insufficient targeted genetic testing focused on a single gene associated with a specific phenotype. It is important that clinicians treating osteodysplastic complex cases, such as the present, promptly pursue genetic counseling and DNA investigation for the patients. Collaboration among scientific groups, in order to compile sets of such complex cases, would be beneficial for the enhanced understanding of phenotype–genotype correlations when multiple genes are involved. This would facilitate the comparison of genotypes and manifestations, ultimately resulting in clearer and more generalizable conclusions regarding genetic interactions in order to identify which genes within the mutated gene set exhibit functional epistasis or hypostasis, which is crucial in mixed phenotypes.

Following the enhanced comprehension of genetic interactions and genotype–phenotype correlations in affected patients, subsequent functional in vitro studies and the development of complex animal models shall not only be of great importance for elucidating both the functional impact of frequently occurring mutations on osteocyte morphology, homeostasis, and function, but also in the revealing of compensatory pathways that may determine and explain the mixed osteodysplastic phenotypes. Having established a map spanning from the effects of single mutations, both in documented patients and in cultured osteocytes, to the mixed genotype induction in animals, could ultimately allow for a deeper understanding of the way that a set of genetic interactions could orchestrate such complex pathologies in cells and subsequently in tissues. Possessing such documented knowledge on that matter might set the ground for the development of future multi-target therapies that could be tested in fully representative genetically engineered experimental animals. The above research steps might pave the way for personalized therapeutic strategies, adapted to the unique genetic profile representing each complex case.

## 5. Conclusions

The present case highlights the rare coexistence of multiple osteodysplastic syndromes in a single patient. The identification of novel pathogenic variants in the *CREB3L1*, *SLCO2A1*, *SFRP4*, and *LRP5* genes adds valuable information to the understanding of these conditions. Molecular modeling analysis and molecular simulation dynamics revealed the disruption of the mutant protein structures and functions caused by the observed pathogenic variants. Furthermore, this case underlines the importance of combinatory diagnosis and emphasizes the necessity for early genetic investigation of such complex cases, with overlying phenotypic traits, followed by genetic counseling, in order to orchestrate clinical interventions and prevent or promptly manage possible future manifestations. Additional studies are needed to explore the full spectrum of phenotypic manifestations associated with the detected variants and to establish clearer genotype–phenotype correlations.

## Figures and Tables

**Table 1:** Shows a summary regarding the variants identified in the patient.

Gene	Variant	Genotype	Protein Role	Potential Effect	Inheritance	Disease Links
*CREB3L1*	c.1523+3G>-	Homozygote	Transcription factor	Abnormal splicing	AR	Osteogenesis imperfecta type XVI
*SLCO2A1*	c.397+10T>C	Homozygote	Membrane anion transporter	Abnormal splicing	AR	Primary hypertrophic osteoarthropathy
*SFRP4*	c.958C>A P320T	Homozygote	Wnt antagonist	May impair Wnt inhibition	AR	Pyle’s Metaphyseal Dysplasia
*LRP5*	c.3184A>G R638H	Heterozygote	Wnt co-receptor	May affect ligand binding or folding	AD	Endosteal Hyperostosis
*LRP6*	c.3184A>G I1062V	Heterozygote	Wnt co-receptor	Conservative change; may subtly impair signaling	AD	Osteoporosis

**Table 2:** Potential clinical effect of variants affecting key components of the Wnt signaling pathway.

Variant	Gene	Protein Role	Potential Effect	Disease Links
P320T	*SFRP4*	Wnt antagonist	May impair Wnt inhibition	Pyle’s Metaphyseal Dysplasia (bone dysplasia)
R638H	*LRP5*	Wnt co-receptor	May affect ligand binding or folding	Endosteal Hyperostosis (increased bone density)
I1062V	*LRP6*	Wnt co-receptor	Conservative change; may subtly impair signaling	Osteoporosis (decreased bone density)

## Data Availability

All related data and materials are available.
